# SeqPig: simple and scalable scripting for large sequencing data sets in Hadoop

**DOI:** 10.1093/bioinformatics/btt601

**Published:** 2013-10-22

**Authors:** André Schumacher, Luca Pireddu, Matti Niemenmaa, Aleksi Kallio, Eija Korpelainen, Gianluigi Zanetti, Keijo Heljanko

**Affiliations:** ^1^Aalto University School of Science and Helsinki Institute for Information Technology HIIT, Finland, ^2^International Computer Science Institute, Berkeley, CA, USA, ^3^CRS4—Center for Advanced Studies, Research and Development in Sardinia, Italy and ^4^CSC—IT Center for Science, Finland

## Abstract

**Summary:** Hadoop MapReduce-based approaches have become increasingly popular due to their scalability in processing large sequencing datasets. However, as these methods typically require in-depth expertise in Hadoop and Java, they are still out of reach of many bioinformaticians. To solve this problem, we have created SeqPig, a library and a collection of tools to manipulate, analyze and query sequencing datasets in a scalable and simple manner. SeqPigscripts use the Hadoop-based distributed scripting engine Apache Pig, which automatically parallelizes and distributes data processing tasks. We demonstrate SeqPig’s scalability over many computing nodes and illustrate its use with example scripts.

**Availability and Implementation:** Available under the open source MIT license at http://sourceforge.net/projects/seqpig/

**Contact:** andre.schumacher@yahoo.com

**Supplementary information:** Supplementary data are available at *Bioinformatics* online.

## 1 INTRODUCTION

Novel computational approaches are required to cope with the increasing data volumes of large-scale sequencing projects, as the growth in processing power and storage access speed is unable to keep pace with them (Marx, 2013; Stein, 2010). Several innovative tools and technologies have been proposed to tackle these challenges. Some are based on MapReduce, which is a distributed computing paradigm that is based on the idea of splitting input data into chunks, which can be processed largely independently (via a *Map* function). Subresults can later be merged after grouping-related subresults (by a *Reduce* function). MapReduce permits automatic parallelization and scalable data distribution across many computers. The most popular implementation available as open-source software is Apache Hadoop, which also comes with its own distributed file system. The validity of Hadoop as a data processing platform is demonstrated by the level of adoption in major data-intensive companies, e.g. Twitter, Facebook and Amazon.

Motivated by the potential scalability and throughput offered by Hadoop, there are an increasing number of Hadoop-based tools for processing sequencing data (Taylor, 2010), ranging from quality control (Robinson *et al.*, 2011) and alignment (Langmead *et al.*, 2009; Pireddu *et al.*, 2011) to SNP calling (Langmead *et al.*, 2009), variant annotation (O’Connor *et al.*, 2010) and structural variant detection (Whelan *et al.*, 2013), including general purpose workflow management (Schönherr *et al.*, 2012). Note the recent publication of independent and complimentary work in (Nordberg *et al.*, 2013).

Although Hadoop does simplify writing scalable distributed software, it does not make it trivial. Such a task still requires specialized skills and a significant amount of work, particularly if the solution involves sequences of MapReduce jobs. This effort can be reduced significantly by using high-level tools such as Apache Pig, which implements an SQL-like scripting language that is automatically translated into a sequence of MapReduce jobs. Given its flexibility and simplicity for developing data processing pipelines, it is not surprising that a large fraction of computing jobs in contemporary Hadoop deployments originate from Apache Pig or similar high-level tools (Chen *et al.*, 2012). SeqPig brings the benefits of Apache Pig to sequencing data analysis. It allows users to integrate their own analysis components with existing MapReduce programs to create full NGS pipelines based on Hadoop.

## 2 METHODS

SeqPig extends Pig with a number of features and functionalities specialized for processing sequencing data. Specifically, it provides (i) data input and output components, (ii) functions to access fields and transform data and (iii) a collection of scripts for frequent tasks (e.g. pile-up, QC statistics).

Apache Pig provides an extension mechanism through the definition of new library functions, implemented in one of several supported programming languages (Java, Python, Ruby, JavaScript); these functions can then be called from Pig scripts. SeqPig uses this feature to augment the set of operators provided by plain Pig with a number of custom sequencing-specific functions.

**Fig. 1.**
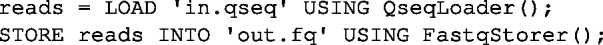
Converting Qseq into Fastq; the dataset is simply read and then written using the appropriate load/store functions

**Fig. 2.**
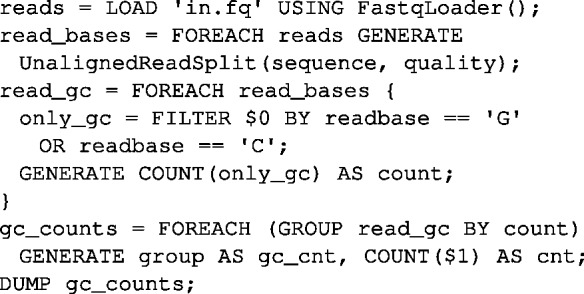
Script that generates a histogram for GC content of reads. The script loads a Fastq file, splits each read into separate bases and for each read coordinate filters only bases that are either G or C. The filtered bases are then counted and counts are grouped. Finally, the script prints records which contain the GC count and the count of reads that have the given GC count

SeqPig supports *ad hoc* (scripted and interactive) distributed manipulation and analysis of large sequencing datasets so that processing speed scales with the number of available computing nodes. It provides import and export functions for file formats commonly used for sequencing data: Fastq, Qseq, FASTA, SAM and BAM. These components, implemented with the help of Hadoop-BAM (Niemenmaa *et al.*, 2012), allow the user to load and export sequencing data in the Pig environment. All available fields, such as BAM/SAM optional read attributes, for example, can then be accessed and modified from within Pig. SeqPig also includes functions to access SAM flags, split reads by base (for computing base-level statistics), reverse-complement reads, calculate read reference positions in a mapping (for pileups, extracting SNP positions) and more. It comes packaged with scripts that calculate various statistics and manipulations on read data, which also serve as examples. The growing library of functions and scripts is documented in the SeqPig manual. Contributions from the community are welcome and encouraged. Figures 1 and 2 show script examples. For a more detailed list of features and more examples please see the project Web site: http://seqpig.sourceforge.net/.

To evaluate SeqPig, we implemented a script that calculates most of the read quality statistics that are collected by the popular FastQC tool (Andrews, 2010). The script is included with the examples (fast_fastqc.pig). We ran a set of experiments, which measured the speed-up gained by using SeqPig on Hadoop clusters of different sizes compared with using a single-node FastQC run. We used a set of Illumina reads as input (read length: 101 bases; file size: 61.4 GB; format: Fastq). Software versions were as follows: FastQC 0.10.1; Hadoop 1.0.4; Pig 0.11.1. All tests were run on nodes equipped with dual quad-core Intel Xeon CPUs @ 2.83 GHz, 16 GB of RAM and one 250 GB SATA disk available to Hadoop. Nodes are connected via Gigabit Ethernet. FastQC read its data from a high-performance shared parallel file system by DDN. SeqPig used the Hadoop file system, which uses each node’s local disk drive.

**Fig. 3.**
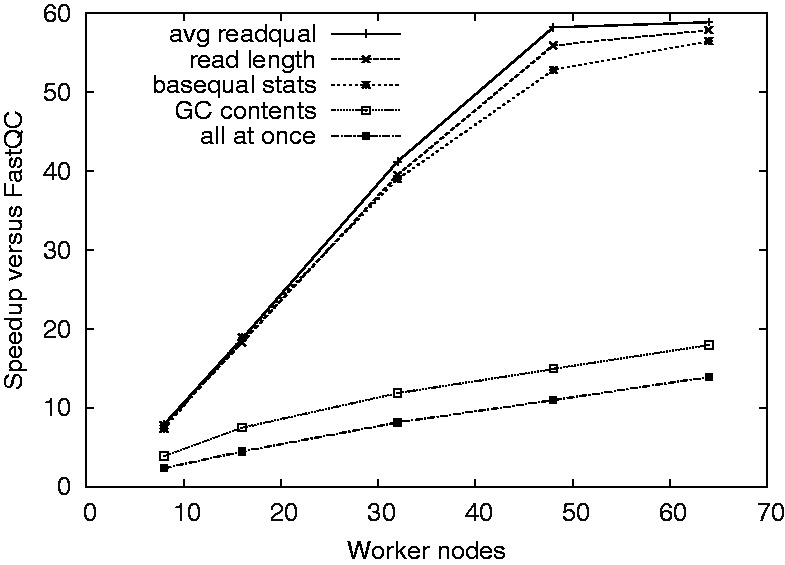
Results of an experiment with an input file of 61.4 GB and a different number of Hadoop worker nodes. The script does not currently implement all FastQC statistics (we expect the missing ones to scale similarly in SeqPig), whereas the per-cycle quality distribution is not computed by FastQC

We first ran five different SeqPig read statistics for a different number of computing nodes: the sample distribution of (i) the average base quality of the reads; (ii) the length of reads; (iii) bases by position inside the reads; and (iv) the GC contents of reads. Finally, we combined them into a single script. Each of the executions results in a single MapReduce job and thus a single scan through the data. All runs were repeated three times and averaged (deviation from average <7%). From Figure 3 one can see that it is possible to achieve a significant speed-up by exploiting the parallelism in read and base statistics computation using Hadoop. Further, the total runtime of the script that computes all statistics is mostly determined by the slowest of the individual ones, as the complete script is compiled into a single map-only job. A different observation is that for most of the statistics computed, we are able to achieve a close to linear speed-up compared with FastQC until 48 nodes. We assume that the leveling off is due to the Hadoop job overhead eventually dominating over speed-up due to parallelization, depending on input file size.

SeqPig enables simple and scalable manipulation and analysis of sequencing data on the Hadoop platform. At CRS4 SeqPig is already used routinely for several steps in the production workflow; in addition, it has been successfully used for *ad hoc* investigations into data quality issues, comparison of alignment tools and reformatting and packaging data. We have also tested SeqPig on Amazon’s Elastic MapReduce service, where users may rent computing time on the cloud to run their SeqPig scripts and even share their S3 storage buckets with other cloud-enabled software. Instructions are provided in the Supplementary Material.

## Supplementary Material

Supplementary Data

## Notes

*Funding*: This work was supported by the Cloud Software and D2I Programs of the Finnish Strategic Centre for Science, Technology and Innovation DIGILE; Academy of Finland [139402]; the Sardinian (Italy) Regional Grant [L7-2010/COBIK]; COST Action BM1006 “Next Generation Sequencing Data Analysis Network”, SeqAhead. Computational resources were provided by CRS4 and ELIXIR node hosted at CSC–IT Center for Science.
