# Supplementary material for: SeqPig: simple and scalable scripting for large sequencing data sets in Hadoop
Source: Bioinformatics. 2013 Oct 22;30(1):119–20. doi: 10.1093/bioinformatics/btt601 (PMC3866557; doi:10.1093/bioinformatics/btt601)
Supplement: Supplementary Data [file supp_30_1_119__index.html]

SeqPig: simple and scalable scripting for large sequencing data sets in Hadoop — SeqPig: simple and scalable scripting for large sequencing data sets in Hadoop — SeqPig: simple and scalable scripting for large sequencing data sets in Hadoop — Supplementary Data 

# SeqPig: simple and scalable scripting for large sequencing data sets in Hadoop

## Supplementary Data

files

**Files in this Data Supplement:**

- Supplementary Data - pdf file
