# Supplementary material for: SeqPig: simple and scalable scripting for large sequencing data sets in Hadoop
Source: Bioinformatics. 2013 Oct 22;30(1):119–20. doi: 10.1093/bioinformatics/btt601 (PMC3866557; doi:10.1093/bioinformatics/btt601)
Supplement: Supplementary Data [file supp_btt601_supplement.pdf]

SeqPig: simple and scalable scripting for large sequencing data sets in Hadoop

Supplementary material

1 Introduction

SeqPig is a library for Apache Pig `http://pig.apache.org/` for the distributed analysis of large sequencing datasets on Hadoop clusters. With SeqPig one can easily take advantage of the advanced high-level features of Pig to manipulate and analyze sequencing data, thus writing simple scripts that automatically run as scalable distributed programs on potentially very large Hadoop clusters.

SeqPig provides a number of features and functionalities. It provides import and export functions for file formats commonly used in bioinformatics, as well as a collection of Pig user-defined-functions (UDF's) specifically designed for processing aligned and unaligned sequencing data. Currently SeqPig supports BAM, SAM, FastQ and Qseq input and output and FASTA input, thanks in part to the functionality provided by the Hadoop-BAM library.

Releases of SeqPig come bundled with Picard/Samtools, which is developed at the Wellcome Trust Sanger Institute, and Seal, which is developed at CRS4. For more details on these projects see their respective web sites, at `http://picard.sourceforge.net/`, `http://samtools.sourceforge.net/` and `http://biodoop-seal.sourceforge.net/`.

This supplementary material consists of three parts. The first part describes installation and dependencies, while the second part discusses a number of example use cases and describes their implementation as SeqPig scripts. In the final section we discuss some Hadoop and Pig parameters that may be changed for improvements in runtime and resource utilization.

2 Installation

Since SeqPig builds on top of Pig, which itself relies on Hadoop for job execution, the installation requires a working Pig and Hadoop setup. For more information in this direction see for example `http://pig.apache.org/docs/r0.11.1/start.html`. A convenient way to install Hadoop is provided by the the Cloudera Hadoop distribution (CDH).

2.1 Dependencies

We have tested SeqPig with the following dependencies.

- Hadoop versions 0.20.2 or 1.0.4
- Pig (at least version 0.10)

The following two dependencies are already included in a pre-compiled SeqPig release:

- Hadoop-BAM (`https://sourceforge.net/projects/hadoop-bam/`)
- Seal (`http://biodoop-seal.sourceforge.net/`)

2.2 Environment variables

- Set Hadoop-related variables (e.g., HADOOP_HOME) for your installation
- Set PIG_HOME to point to your Pig installation

On a Cloudera Hadoop installation with Pig a suitable environment configuration would be:

```
$ export HADOOP_HOME=/usr/lib/hadoop
$ export PIG_HOME=/usr/lib/pig
```

2.3 Installing a pre-compiled release

1. Dowload the latest SeqPig release from `http://sourceforge.net/projects/seqpig/files/`:

   ```
   $ wget http://sourceforge.net/projects/seqpig/files/seqpig_0.5.tar.gz
   ```

2. Untar the release into an installation directory of your choice and set the SEQPIG_HOME environment variable to point to the installation directory of SeqPig; e.g.,

   ```
   $ tar xvzf seqpig_0.5.tar.gz -C /usr/local/java/
   $ export SEQPIG_HOME=/usr/local/java/seqpig
   ```

3. For your convenience, you can add the bin directory to your PATH:

   ```
   $ export PATH=${PATH}:${SEQPIG_HOME}/bin
   ```

   This way, you'll be able to start a SeqPig-enabled Pig shell by running the `seqpig` command.

Note that since the sources are included in the release you are also able to build SeqPig if necessary, as described below.

2.4 Instructions for building SeqPig

1. Download Hadoop-BAM 5.1 from `http://sourceforge.net/projects/hadoop-bam/files/`.

   ```
   $ wget http://sourceforge.net/projects/hadoop-bam/files/hadoop-bam-5.1.tar.gz
   $ tar xvzf hadoop-bam-5.1.tar.gz
   $ export HADOOP_BAM=`pwd`/hadoop-bam-5.1
   ```

2. Download and build the latest Seal git master version from `http://biodoop-seal.sourceforge.net/`. Note that this requires setting HADOOP_BAM to the installation directory of Hadoop-BAM, as done in the previous step.

   ```
   $ git clone git://git.code.sf.net/p/biodoop-seal/code biodoop-seal-code
   ```

   For more information on how to build Seal see `http://biodoop-seal.sourceforge.net/installation.html`.

3. Clone the SeqPig repository.

   ```
   $ git clone git://git.code.sf.net/p/seqpig/code seqpig-code
   ```

4. Inside the cloned SeqPig git repository create a `lib/` subdirectory and copy (or link) the jar files from Hadoop-BAM and Seal to this new directory. The files should be:

   a) `${HADOOP_BAM}/*.jar`

   b) from the Seal directory, run `find build/ -name seal.jar`

   Note: the Picard and Sam jar files are contained in the Hadoop-BAM release for convenience.

5. Run `ant` to build `SeqPig.jar`.

Once you've built SeqPig, you can move the directory to a location of your preference (if on a shared system, perhaps /usr/local/java/seqpig, else even your home directory could be fine).

2.4.1 Note

Some of the example scripts in this manual (e.g., Section 3.2.6) require functions from *PiggyBank*, which is a collection of publicly available User-Defined Functions (UDF's) that are distributed with Pig but may need to be built separately, depending on your Pig distribution. For more details see https://cwiki.apache.org/confluence/display/PIG/PiggyBank. Verify that PiggyBank has been compiled by looking for the file piggybank.jar under $PIG_HOME:

```
$ find $PIG_HOME -name piggybank.jar
```

If PiggyBank hasn't been compiled, go into $PIG_HOME/contrib/piggybank/java and run ant.

2.5 Running on Amazon Elastic MapReduce

Assuming you have started an interactive Pig Job Flow (for example via the AWS console), you can login into the master node and copy the SeqPig release to the Hadoop user home directory. Then set both SEQPIG_HOME and PIG_HOME correctly (HADOOP_HOME should be set by default). Note that the Pig version installed does not necessarily match the latest Pig release. The advantage, however, is the ability to use S3 buckets for input and output.

Consider the following example for starting SeqPig on Amazon Elastic MapReduce. In this example we install SeqPig into /home/hadoop/seqpig.

```
$ wget http://sourceforge.net/projects/seqpig/files/seqpig_0.5.tar.gz
$ tar -C /home/hadoop -xvzf seqpig_0.5.tar.gz
$ export SEQPIG_HOME=/home/hadoop/seqpig
$ export PIG_HOME=/home/hadoop/.versions/pig-0.9.2
$ /home/hadoop/seqpig/bin/seqpig
```

2.6 Tests

After building SeqPig it may be a good idea to run tests to verify that the environment has been set up correctly. When inside the SeqPig directory execute

```
$ test/test_all.sh
```

By default the tests run Pig in local mode. In order to test Hadoop mode pass the command line argument -h. Note that this test requires that Hadoop to be set up correctly. It first imports a BAM file, sorts the reads by coordinate and converts it to SAM for comparing the results. The test should end with the line

```
TEST: all tests passed
```

If you intend to run SeqPig on an Amazon Elastic MapReduce instance, you can also test input from S3 by providing an S3 path to the file data/input.bam:

```
$ test/test_all.sh -s <s3_path>
```

for example:

```
$ test/test_all.sh -s seqpig/data/input.bam
```

where seqpig is the name of the S3 bucket.

2.7 Usage

2.7.1 Pig grunt shell for interactive operations

Assuming that all the environment variables have been set as described in the previous sections, you can start the SeqPig-enabled "grunt" shell by running

```
$ seqpig
```

If you prefer to tun SeqPig in local mode (without Hadoop), which can be useful for debugging scripts, you can start it by running

```
$ seqpig -x local
```

2.7.2 Starting scripts from the command line for non-interactive use

Alternatively to using the interactive Pig grunt shell, users can write scripts that are then submitted to Pig/Hadoop for automated execution. This type of execution has the advantage of being able to handle parameters; for instance, one can parametrize input and output files. See the `/scripts` directory inside the SeqPig distribution and Section 3 for examples.

3 Examples

This section lists a number of examples of the types of operations that can be performed with SeqPig. Each example is typically a mix of standard Pig operations and SeqPig user-defined functions (UDF's). Note that once the data has been imported to Pig the only difference between, say, read data originating from BAM and Fastq files, is that some fields in tuples derived from BAM may not be available in those from Fastq, e.g., alignment start positions.

3.1 Operations on BAM files

To access sequencing data in BAM files, SeqPig uses Hadoop-BAM, which provides access to all fields and optional attributes in the data records. All the examples below assume that an input BAM file is initially imported to HDFS via

```
$ prepareBamInput.sh input.bam
```

This additional setup step is required to extract the header and store it inside HDFS to simplify some operations that only operate on a chunk of the BAM file without having access to the original header. Similarly, for uncompressed SAM files there is a corresponding `prepareSamInput.sh`. Once the input file is imported into HDFS it can be loaded in the grunt shell via

```
A = load 'input.bam' using BamLoader('yes');
```

The 'yes' parameter to `BamLoader` chooses read attributes to be loaded; choose 'no' whenever these are not required, which is also the default value).

Once some operations have been performed, the resulting (possibly modified) read data can then be stored into a new BAM file via

```
store A into 'output.bam' using BamStorer('input.bam.asciiheader');
```

and can also be exported from HDFS to the local filesystem via

```
prepareBamOutput.sh output.bam
```

Note The Pig store operation requires a valid header for the BAM output file, for example the header of the source file used to generate it, which is generated automatically by the `prepareBamInput.sh` script used to import it).

3.1.1 Simple operations

Writing the BAM data to the screen (similarly to `samtools view`) can be done simply by

```
dump A;
```

Another very useful Pig command is `describe`, which returns the schema that Pig uses for a given data bag. Example:

```
A = load 'input.bam' using BamLoader('yes');
describe A;
```

returns

```
 A: {name: chararray,start: int,end: int,read: chararray,cigar: chararray,
  basequal: chararray,flags: int,insertsize: int,mapqual:int,matestart: int,
  materefindex: int,refindex: int,refname: chararray,attributes: map[]}
```

Notice that all fields except the attributes are standard data types (strings or integers). Specific attributes can be accessed via `attributes#'name'`. For example,

```
B = FOREACH A GENERATE name, attributes#'MD';
dump B;
```

will output all read names and their corresponding MD tag. Other useful commands are `LIMIT` and `SAMPLE`, which can be used to select a subset of reads from a BAM/SAM file.

```
B = LIMIT A 20;
```

will assign the first 20 records of A to B, while

```
B = SAMPLE A 0.01;
```

will sample from A with sampling probability 0.01.

3.1.2 Filtering out unmapped reads and PCR or optical duplicates

Since the `flags` field of a SAM record is exposed to Pig, one can simply use it to filter out all tuples (i.e., SAM records) that do not have the corresponding bit set.

```
A = FILTER A BY (flags/4)%2==0 and (flags/1024)%2==0;
```

For convenience SeqPig provides a set of filters that allow a direct access to the relevant fields. The previous example is equivalent to

```
run scripts/filter_defs.pig
A = FILTER A BY not ReadUnmapped(flags) and not IsDuplicate(flags);
```

Note that the above example assumes that the Pig shell was started in the SeqPig root directory. If this is not the case, you need to adjust the path to `filter_defs.pig` accordingly. For of a full list of the available filters look at this file.

3.1.3 Filtering out reads with low mapping quality

Other fields can also be used for filtering, for example the read mapping quality value as shown below.

```
A = FILTER A BY mapqual > 19;
```

3.1.4 Filtering by regions (samtools syntax)

SeqPig also supports filtering by samtools *region* syntax. The following examples selects base positions 1 to 44350673 of chromosome 20.

```
DEFINE myFilter CoordinateFilter('input.bam.asciiheader','20:1-44350673');
A = FILTER A BY myFilter(refindex,start,end);
```

Note that filtering by regions requires a valid SAM header for mapping sequence names to sequence indices. This file is generated automatically when BAM files are imported via the `prepareBamInput.sh` script.

3.1.5 Sorting BAM files

Sorting an input BAM file by chromosome, reference start coordinate, strand and readname (in this hierarchical order):

```
A = FOREACH A GENERATE name, start, end, read, cigar, basequal, flags, insertsize,
mapqual, matestart, materefindex, refindex, refname, attributes, (flags/16)%2 AS strand;
A = ORDER A BY refname, start, strand, name;
```

Note that `strand` here is used to refer to the strand flag computed by the expression `(flags/16)%2`.

Note This is roughly equivalent to executing from the command line:

```
$ seqpig -param inputfile=input.bam -param outputfile=input_sorted.bam \
  ${SEQPIG_HOME}/scripts/sort_bam.pig
```

3.1.6 Computing read coverage

Computing read coverage over reference-coordinate bins of a fixed size, for example:

```
B = GROUP A BY start/200;
C = FOREACH B GENERATE group, COUNT(A);
dump C;
```

will output the number of reads that lie in any non-overlapping bin of size 200 base pairs. For a more precise coverage computation see Section 3.1.8 on computing read pileup.

3.1.7 Computing base frequencies (counts) for each reference coordinate

```
run scripts/filter_defs.pig
A = FOREACH A GENERATE read, flags, refname, start, cigar, basequal, mapqual;
A = FILTER A BY not ReadUnmapped(flags);
RefPos = FOREACH A GENERATE ReadRefPositions(read, flags, refname, start, cigar, basequal),
    mapqual;
flatset = FOREACH RefPos GENERATE flatten($0), mapqual;
grouped = GROUP flatset BY ($0, $1, $2);
base_counts = FOREACH grouped GENERATE group.chr, group.pos, group.base, COUNT(flatset);
base_counts = ORDER base_counts BY chr,pos;
store base_counts into 'input.basecounts';
```

Note This is roughly equivalent to executing from the command line:

```
$ seqpig -param inputfile=input.bam -param outputfile=input.basecounts \
  -param pparallel=1 ${SEQPIG_HOME}/scripts/basefreq.pig
```

3.1.8 Pileup

Generating samtools compatible pileup (for a correctly sorted BAM file with MD tags aligned to the same reference, should produce the same output as `samtools mpileup -A -f ref.fasta -B input.bam`):

```
run scripts/filter_defs.pig
A = load 'input.bam' using BamLoader('yes');
B = FILTER A BY not ReadUnmapped(flags) and not IsDuplicate(flags);
C = FOREACH B GENERATE ReadPileup(read, flags, refname, start, cigar,
     basequal, attributes#'MD', mapqual), start, flags, name;
C = FILTER C BY $0 is not null;
D = FOREACH C GENERATE flatten($0), start, flags, name;
E = GROUP D BY (chr, pos);
F = FOREACH E {
  G = FOREACH D GENERATE refbase, pileup, qual, start, (flags/16)%2, name;
  G = ORDER G BY start, $4, name;
  GENERATE group.chr, group.pos, PileupOutputFormatting(G, group.pos);
}
G = ORDER F BY chr, pos;
H = FOREACH G GENERATE chr, pos, flatten($2);
store H into 'input.pileup' using PigStorage('\t');
```

Note This is equivalent to executing from the command line:

```
$ seqpig -param inputfile=input.bam -param outputfile=input.pileup -param pparallel=1 \
  ${SEQPIG_HOME}/scripts/pileup.pig
```

The script essentially does the following:

1. Import BAM file and filter out unmapped or duplicate reads (`A, B`)
2. Break up each read and produce per-base pileup output (`C, D`)
3. Group all thus generated pileup output based on a (chromosome, position) coordinate system (`E`)
4. For each of the groups, sort its elements by their position, strand and name; then format the output according to samtools (`F`)
5. Sort the final output again by (chromosome, position) and flatten it to un-nest the tuples (`G, H`)
6. Store the output to a directory inside HDFS (last line)

There are two optional parameters for `pileup.pig`: `min_map_qual` and `min_base_qual` (both with default value 0) that filter out reads with either insufficient map quality or base qualities. Their values can be set the same way as the other parameters above.

There is an alternative pileup script which typically performs better but is more sensitive to additional parameters. This second script, `pileup2.pig`, is based on a *binning* of the reads according to intervals on the reference sequence. The pileup output is then generated on a by-bin level and not on a by-position level. This script can be invoked with the same parameters as `pileup2.pig`. However, it has tunable parameters that determine the size of the bins (`binsize`) and the maximum number of reads considered per bin (`reads_cutoff`), which is similar to the maximum depth parameter that samtools accepts. However, note that since this parameter is set on a per-bin level you may choose it dependent on the read length and bin size, as well as the amount of memory available on the compute nodes.

3.1.9 Collecting read-mapping-quality statistics

In order to evaluate the output of an aligner, it may be useful to consider the distribution of the mapping quality over the collection of reads. Thanks to Pig's `GROUP` operator this is fairly easy.

```
run scripts/filter_defs.pig
A = load 'input.bam' using BamLoader('yes');
B = FILTER A BY not ReadUnmapped(flags) and not IsDuplicate(flags);
read_stats_data = FOREACH B GENERATE mapqual;
read_stats_grouped = GROUP read_stats_data BY mapqual;
read_stats = FOREACH read_stats_grouped GENERATE group, COUNT($1);
read_stats = ORDER read_stats BY group;
STORE read_stats into 'mapqual_dist.txt';
```

Note This is equivalent to executing from the command line:

```
$ seqpig -param inputfile=input.bam -param outputfile=mapqual_dist.txt \
  ${SEQPIG_HOME}/scripts/read_stats.pig
```

3.1.10 Collecting per-base statistics of reads

Sometimes it may be useful to analyze a given set of reads for a bias towards certain bases being called at certain positions inside the read. The following simple script generates for each reference base and each position inside a read the distribution of the number of read bases that were called.

```
run scripts/filter_defs.pig
A = load 'input.bam' using BamLoader('yes');
B = FILTER A BY not ReadUnmapped(flags) and not IsDuplicate(flags);
C = FOREACH B GENERATE ReadSplit(name,start,read,cigar,basequal,flags,mapqual,refindex,
    refname,attributes#'MD');
D = FOREACH C GENERATE FLATTEN($0);
base_stats_data = FOREACH D GENERATE refbase, basepos, UPPER(readbase) AS readbase;
base_stats_grouped = GROUP base_stats_data BY (refbase, basepos, readbase);
base_stats_grouped_count = FOREACH base_stats_grouped GENERATE group.$0 AS refbase, group.$1
    AS basepos, group.$2 as readbase, COUNT($1) AS bcount;
base_stats_grouped = GROUP base_stats_grouped_count by (refbase, basepos);
base_stats = FOREACH base_stats_grouped {
       TMP1 = FOREACH base_stats_grouped_count GENERATE readbase, bcount;
       TMP2 = ORDER TMP1 BY bcount desc;
       GENERATE group.$0, group.$1, TMP2;
   }
STORE base_stats into 'outputfile_readstats.txt';
```

Here is an example output (for a BAM file with 50 reads):

```
A      0      {(A,19),(G,2)}
A      1      {(A,10)}
A      2      {(A,18)}
A      3      {(A,16)}
A      4      {(A,14)}
A      5      {(A,15)}
A      6      {(A,16),(G,2)}
...
A      98     {(A,7)}
A      99     {(A,14)}
C      0      {(C,6)}
C      1      {(C,11)}
```

```
C       2       {(C,9)}
...
```

Note This example script is equivalent to executing from the command line:

```
$ seqpig -param inputfile=input.bam -param outputfile=outputfile_readstats.txt \
  $SEQPIG_HOME/scripts/basequal_stats.pig
```

3.1.11 Collecting per-base statistics of base qualities for reads

Analogously to the previous example collecting statistics for the read bases, we can also collect frequencies for base qualities conditioned on the position of the base inside the reads. If these fall off too quickly for later positions, it may indicate some quality issues with the run. The resulting script is actually fairly similar to the previous one with the difference of not grouping over the reference bases.

```
run scripts/filter_defs.pig
A = load 'input.bam' using BamLoader('yes');
B = FILTER A BY not ReadUnmapped(flags) and not IsDuplicate(flags);
C = FOREACH B GENERATE ReadSplit(name,start,read,cigar,basequal,flags,mapqual,refindex,
    refname,attributes#'MD');
D = FOREACH C GENERATE FLATTEN($0);
base_stats_data = FOREACH D GENERATE basepos, basequal;
base_stats_grouped = GROUP base_stats_data BY (basepos, basequal);
base_stats_grouped_count = FOREACH base_stats_grouped GENERATE group.$0 as basepos, group.$1
    AS basequal, COUNT($1) AS qcount;
base_stats_grouped = GROUP base_stats_grouped_count BY basepos;
base_stats = FOREACH base_stats_grouped {
       TMP1 = FOREACH base_stats_grouped_count GENERATE basequal, qcount;
       TMP2 = ORDER TMP1 BY basequal;
       GENERATE group, TMP2;
}
STORE base_stats into 'outputfile_basequalstats.txt';
```

Here is an example output (for a BAM file with 50 reads):

```
0       {(37,10),(42,1),(51,20),(52,1),(59,1),(61,1),(62,1),(67,2),(68,2),(70,2),(71,4),(72,3)
    ,(73,1),(75,2)}
1       {(53,1),(56,1),(61,1),(63,1),(64,1),(65,2),(67,4),(68,3),(69,2),(70,7),(71,3),(72,3)
    ,(73,1),(74,4),(75,2),(76,5),(77,6),(78,2),(80,1)}
2       {(45,1),(46,1),(51,2),(57,1),(61,1),(65,2),(66,3),(67,2),(69,3),(71,4),(72,2),(73,6)
    ,(74,7),(75,1),(76,8),(77,2),(78,3),(80,1)}
3       {(58,1),(59,1),(60,1),(61,1),(62,1),(64,1),(65,2),(67,2),(68,1),(69,5),(70,1),(71,3)
    ,(72,7),(73,2),(74,4),(75,6),(76,2),(77,4),(78,3),(79,1),(81,1)}
4       {(55,1),(60,1),(61,1),(62,1),(64,1),(66,1),(67,3),(68,2),(69,1),(70,7),(71,2),(72,1)
    ,(73,4),(74,2),(75,2),(76,2),(77,2),(78,3),(79,7),(80,4),(81,2)}
5       {(51,1),(52,2),(54,1),(58,2),(62,2),(63,1),(66,3),(68,4),(70,1),(71,1),(72,2),(73,3)
    ,(74,1),(75,8),(76,1),(77,5),(78,1),(79,6),(80,3),(81,3)}
...
```

Note This example script is equivalent to executing from the command line:

```
$ seqpig -param inputfile=input.bam -param outputfile=outputfile_basequalstats.txt \
  $SEQPIG_HOME/scripts/basequal_stats.pig
```

3.1.12 Filtering reads by mappability threshold

The script filter_mappability.pig filters reads in a given BAM file based on a given mappability threshold. Both input BAM and mappability file need to reside inside HDFS.

```
$ seqpig -param inputfile=/user/hadoop/input.bam -param outputfile=/user/hadoop/output.bam \
  -param regionfile=/user/hadoop/mappability.100kbp.txt -param threshold=90 \
  $SEQPIG_HOME/scripts/filter_mappability.pig
```

Note that since the script relies on distributing the BAM file header and the mappability file via Hadoop's distributed cache, it is not possible to run it with Pig in local mode.

3.2 Processing Qseq and Fastq data

SeqPig supports the import and export of non-aligned reads stored in Qseq and Fastq data. Due to Pig's model that all records correspond to tuples, which form bags, reads can be processed in very much the same way independent of, for example, whether they are stored in Qseq or Fastq. Also, since Fastq and Qseq files are header-less, there is no pre-processing step required prior to loading the files, compared to SAM/BAM import.

3.2.1 Converting Qseq to Fastq and vice versa

The following two lines simply convert an input Qseq file into Fastq format.

```
reads = load 'input.qseq' using QseqLoader();
STORE reads INTO 'output.fastq' using FastqStorer();
```

The other direction works analogously.

The following examples are not specific to the type of input data (Fastq, Qseq, BAM, ...). They roughly correspond to the ones computed by the popular FastQC tool and can be all found in the fast_fastqc.pig script.

3.2.2 Computing a read length distribution

Once a read set is imported, it is fairly straightforward to compute simple statistics, such as a histogram of the length of reads. Using the LENGTH string function, which is part of the PiggyBank (see Section 2.4.1), we can first compute the set of read lengths, group them by their value and then compute the size of each of the groups.

```
DEFINE LENGTH org.apache.pig.piggybank.evaluation.string.LENGTH();
reads = load 'input.fq' using FastqLoader();
read_len = FOREACH reads GENERATE LENGTH(sequence);
read_len_counts = FOREACH (GROUP read_len BY $0) GENERATE group AS len, COUNT_STAR($1) as
    count;
```

3.2.3 Computing a distribution of the average base quality of a read

By using a SeqPig UDF to split reads into bases, one can compute this statistic by relying on Pig's GROUP BY operator as follows.

```
reads = load 'input.fq' using FastqLoader();
reads_by_bases = FOREACH reads GENERATE UnalignedReadSplit(sequence, quality);
read_q = FOREACH reads_by_bases GENERATE ROUND(AVG($0.basequal)) as read_qual;
read_q_counts = FOREACH (GROUP read_q BY read_qual) GENERATE group as avg_read_qual,
    COUNT_STAR($1) as count;
```

Alternatively, SeqPig also provides another UDF that has better performance for larger read sets.

```
reads = load 'input.fq' using FastqLoader();
read_seq_qual = FOREACH reads GENERATE quality;
avgbase_qual_counts = FOREACH (GROUP read_seq_qual ALL) GENERATE AvgBaseQualCounts($1.$0);
formatted_avgbase_qual_counts = FOREACH avgbase_qual_counts GENERATE
   FormatAvgBaseQualCounts($0);
```

3.2.4 Distribution of GC content over the read set

The following example shows how one can exploit Pig's GROUP and COUNT operators to obtain statistics of the composition of read bases.

```
reads = load 'input.fq' using FastqLoader();
reads_by_bases = FOREACH reads GENERATE UnalignedReadSplit(sequence, quality);
read_gc = FOREACH reads_by_bases {
  only_gc = FILTER $0 BY readbase == 'G' OR readbase == 'C';
  GENERATE COUNT(only_gc) as count;
}
read_gc_counts = FOREACH (GROUP read_gc BY count) GENERATE group as gc_count, COUNT_STAR($1)
    as count;
```

3.2.5 Computing base statistics over the read set

Similarly to GC content, one can rely on Pig operations to compute for each read position the distribution of nucleotides and their base qualities, as given by the aligner. However, in order to improve performance for larger data sets, SeqPig also provides UDF's for this purpose.

```
reads = load 'input.fq' using FastqLoader();
read_seq_qual = FOREACH reads GENERATE sequence, quality;
base_qual_counts = FOREACH (GROUP read_seq_qual ALL) GENERATE BaseCounts($1.$0),
    BaseQualCounts($1.$1);
formatted_base_qual_counts = FOREACH base_qual_counts GENERATE FormatBaseCounts($0),
    FormatBaseQualCounts($1);
base_gc_counts = FOREACH base_qual_counts GENERATE FormatGCCounts($0);
```

Note All the statistics computed by the examples of Sections 3.2.2–3.2.5 can be executed at once via

```
$ seqpig -param inputpath=input.fq -param outputpath=fast-fastqc-output \
  $SEQPIG_HOME/scripts/fast_fastqc.pig
```

3.2.6 Clipping bases and base qualities

Assuming there were some problems in certain cycles of the sequencer, it may be useful to clip bases from reads. This example removes the last 3 bases and their qualities and stores the data under a new filename. Note that here we rely on the `SUBSTRING` and `LENGTH` string functions, which are part of the PiggyBank (see Section 2.4.1).

```
DEFINE SUBSTRING org.apache.pig.piggybank.evaluation.string.SUBSTRING();
DEFINE LENGTH org.apache.pig.piggybank.evaluation.string.LENGTH();
reads = load 'input.qseq' using QseqLoader();
B = FOREACH A GENERATE instrument, run_number, flow_cell_id, lane, tile, xpos, ypos, read,
    qc_passed, control_number, index_sequence, SUBSTRING(sequence, 0, LENGTH(sequence) - 3)
    AS sequence, SUBSTRING(quality, 0, LENGTH(quality) - 3) AS quality;
store B into 'output.qseq' using QseqStorer();
```

Note This example script is equivalent to executing from the command line:

```
$ seqpig -param inputfile=input.qseq -param outputfile=output.qseq -param backclip=3 \
  $SEQPIG_HOME/scripts/clip_reads.pig
```

4 Performance tuning

4.1 Pig parameters

Recent releases of Pig (such as 0.11.0) have a parameter that enables aggregation of partial results on the mapper side, which greatly improves performances of some of the UDF's described above. Further, another option that is worth considering is to use so-called *schema tuples*. For more information see the Pig documentation. The recommended setting is as follows:

- `set pig.exec.mapPartAgg true;`
- `set pig.exec.mapPartAgg.minReduction 5;`
- `set pig.schematuple on;`

4.2 Hadoop parameters

Some scripts (such as `pileup2.pig`) require an amount of memory that depends on the choice of command line parameters. To tune the performance of such operations on the Hadoop cluster, consider the following Hadoop-specific parameters in `mapred-site.xml`.

- `mapred.tasktracker.map.tasks.maximum`:
  the maximum number simultaneous map tasks per worker node
- `mapred.tasktracker.reduce.tasks.maximum`:
  the maximum number simultaneous reducer tasks per worker node
- `mapred.child.java.opts`:
  Java options passed to child virtual machines at the worker nodes; for instanced, this may be used to configure the Java heap size to 1GB with `-Xmx1000M`

Additionally, it is possible to pass command line parameters (such as mapper and reducer memory limits). For instance, consider the Pig invocation (see `tools/tun_all_pileup2.sh`)

```
${PIG_HOME}/bin/pig -Dpig.additional.jars=${SEQPIG_HOME}/lib/hadoop-bam-5.0.jar:${SEQPIG_HOME
    }/build/jar/SeqPig.jar:${SEQPIG_HOME}/lib/seal.jar:${SEQPIG_HOME}/lib/picard-1.76.jar:${
    SEQPIG_HOME}/lib/sam-1.76.jar -Dmapred.job.map.memory.mb=${MAP_MEMORY} -Dmapred.job.
    reduce.memory.mb=${REDUCE_MEMORY} -Dmapred.child.java.opts=-Xmx${CHILD_MEMORY}M -Dudf.
    import.list=fi.aalto.seqpig -param inputfile=$INPUTFILE -param outputfile=$OUTPUTFILE -
    param pparallel=${REDUCE_SLOTS} ${SEQPIG_HOME}/scripts/pileup2.pig
```

By setting appropriate values for `MAP_MEMORY, REDUCE_MEMORY, CHILD_MEMORY` and for the number of available reduce slots `REDUCE_SLOTS` one may be able to improve performance.

4.3 Compression

For optimal performance and space usage it may be advisable to enable the compression of Hadoop map (and possibly reduce) output, as well as temporary data generated by Pig. Compression with Pig can be enabled by setting properties such as

```
 -Djava.library.path=/opt/hadoopgpl/native/Linux-amd64-64
 -Dpig.tmpfilecompression=true -Dpig.tmpfilecompression.codec=lzo
 -Dmapred.output.compress=true
 -Dmapred.output.compression.codec=org.apache.hadoop.io.compress.GzipCodec
```

on the Pig command line or the Hadoop configuration. Note that currently not all Hadoop compression codecs are supported by Pig. The details regarding which compression codec to use depend are beyond the scope of this manual.

5 UDF reference guide

The aim of this section is to give a brief overview of the available UDF's that come with SeqPig, their input and output schemas and their operation. For more detailed information please consult the source code. Note that some of the usage examples that process BAM files assume that unmapped reads or reads that do not have a valid MD tag have been filtered out (for filters see below).

5.1 `seqpig`

5.1.1 `UnalignedReadSplit`

The UnalignedReadSplit UDF takes a bag of reads (read in by the Fastq or Qseq loaders) and splits them into tuples that correspond to the single bases of each read.

Usage:

```
reads = load 'input.fq' using FastqLoader();
reads_by_bases = FOREACH reads GENERATE UnalignedReadSplit(sequence, quality);
```

	Field	Type	Description
Input schema:	*read*	`chararray`	read bases (sequence)
	basequal	`chararray`	base qualities
Output schema: (bag)	*pos*	`integer`	position in read
	readbase	`chararray`	read base
	basequal	`integer`	base quality

5.1.2 `ReadRefPositions`

The ReadRefPositions UDF takes an aligned read and outputs a bag of tuples, one for each base, that gives the position on the reference sequence this base corresponds to.

Usage:

```
reads = load 'input.bam' using BamLoader('yes');
ref_positions = FOREACH reads GENERATE ReadRefPositions(read, flags, refname,
    start, cigar, basequal);
```

	Field	Type	Description
Input schema:	*read*	`chararray`	read bases (sequence)
	flags	`integer`	SAM flags
	chr	`chararray`	reference name / chromosome
	position	`integer`	mapping position (start)
	cigar	`chararray`	CIGAR string
	basequal	`chararray`	base qualities
Output schema: (bag)	*chr*	`chararray`	reference name / chromosome
	pos	`integer`	position (on reference)
	base	`chararray`	read base
	basequal	`chararray`	base quality

5.1.3 ReadSplit

Similarly to the previous UDF, ReadSplit determines the reference position that a particular base inside a read refers to. Additionally, it also tries to determine the base on the reference in questions by parsing the MD tag (and CIGAR).

Usage:

```
reads = load 'input.bam' using BamLoader('yes');
bases = FOREACH reads GENERATE ReadSplit(name,start,read,cigar,basequal,flags,
    mapqual,refindex,refname,attributes#'MD');
```

	Field	Type	Description
Input schema:	*readname*	chararray	read name
	start	integer	mapping position (start)
	read	chararray	read bases (sequence)
	cigar	chararray	CIGAR string
	basequal	chararray	base qualities
	flags	integer	SAM flags
	mapqual	integer	mapping quality
	refindex	integer	reference index (see BAM/SAM loader)
	chr	chararray	reference name / chromosome (see BAM/SAM loader)
	MD	chararray	MD tag
Output schema: (bag)	*chr*	chararray	reference name / chromosome
	pos	integer	position (on reference)
	refbase	chararray	base on reference (if known, else null)
	readname	chararray	read name
	readlength	integer	read length (possibly clipped)
	mapqual	integer	mapping quality
	flags	integer	SAM flags
	basepos	integer	position in read
	readbase	chararray	base on read
	basequal	chararray	base quality (if applicable, else null)

5.1.4 ReverseComplement

The ReverseComplement UDF takes a read base sequence and returns its reverse complement.

Usage:

```
reads = load 'input.fq' using FastqLoader();
reversed_reads = FOREACH reads GENERATE ReverseComplement(sequence);
```

	Field	Type	Description
Input schema:	*read*	chararray	read bases (sequence)
Output schema:	*reversed_read*	chararray	read bases (reverse-complemented)

5.2 seqpig.io

This package contains import and export functions for the various file formats that are supported. In the case of BAM and SAM files, the file formats make use of all the fields that are available for a Picard sequence record (`SAMRecord`).

5.2.1 BamLoader and SamLoader

The BAM and SAM loader output schemas are identical and both loaders take an optional argument that can take values “yes” and “no”, indicating whether the map of attributes should be populated. Reading in attributes incurs an additional overhead during the import of the data, so choose “no” whenever these are not needed. For a proper definition of the various fields see the SAM file format description available at `http://samtools.sourceforge.net/SAMv1.pdf`.

Usage: `reads = load 'input.bam' using BamLoader('yes');`

Output schema:

Field	Type	Description
name	`chararray`	read name
start	`integer`	start position of alignment
end	`integer`	end position of alignment
read	`chararray`	read bases
cigar	`chararray`	CIGAR string
basequal	`chararray`	base qualities
flags	`integer`	flags
insertsize	`integer`	insert size
mapqual	`integer`	mapping quality
matestart	`integer`	mate start position
materefindex	`integer`	mate reference index
refindex	`integer`	reference index
refname	`chararray`	reference name
attributes	`map`	SAMRecord attributes

5.2.2 `BamStorer` and `SamStorer`

The BAM and SAM storer input schemas are identical and both are essentially equal to the output schema of the corresponding loader functions. Note that the order of the fields inside tuples does not matter, only their field names need to be present. Both loaders require, however, the name of a file that contains a valid SAM header. For details see Section 3.1.

Usage: `store reads into 'output.bam' using BamStorer('input.bam.asciiheader');`

Input schema:

Field	Type	Description
name	`chararray`	read name
start	`integer`	start position of alignment
end	`integer`	end position of alignment
read	`chararray`	read bases
cigar	`chararray`	CIGAR string
basequal	`chararray`	base qualities
flags	`integer`	flags
insertsize	`integer`	insert size
mapqual	`integer`	mapping quality
matestart	`integer`	mate start position
materefindex	`integer`	mate reference index
refindex	`integer`	reference index
refname	`chararray`	reference name
attributes	`map`	SAMRecord attributes

5.2.3 `FastqLoader` and `QseqLoader`

Both loaders for unaligned read file formats Fastq and Qseq essentially provide the same output schema for the tuple field names they produce. Note that some fields that are not present in the input data may remain empty.

Usage: `reads = load 'input.fq' using FastqLoader();`

Output schema:

Field	Type	Description
instrument	`chararray`	instrument identifier
run_number	`integer`	run number
flow_cell_id	`chararray`	flow cell ID
lane	`integer`	lane number
tile	`integer`	tile
xpos	`integer`	XPos
ypos	`integer`	YPos
read	`integer`	read ID
qc_passed	`Boolean`	QC flag
control_number	`integer`	control number
index_sequence	`chararray`	index sequence
sequence	`chararray`	read bases
quality	`chararray`	base qualities

5.2.4 `FastqStorer` and `QseqStorer`

The Fastq and Qseq storer input schemas are identical and both are essentially equal to the output schema of the corresponding loader functions. Note that the order of the fields inside tuples does not matter, only their field names need to be present. All fields except *sequence* and *quality* are optional.

Usage: `store reads into 'output.fastq' using FastqStorer();`

Input schema:

Field	Type	Description
instrument	`chararray`	instrument identifier
run_number	`integer`	run number
flow_cell_id	`chararray`	flow cell ID
lane	`integer`	lane number
tile	`integer`	tile
xpos	`integer`	XPos
ypos	`integer`	YPos
read	`integer`	read ID
qc_passed	`Boolean`	QC flag
control_number	`integer`	control number
index_sequence	`chararray`	index sequence
sequence	`chararray`	read bases
quality	`chararray`	base qualities

5.2.5 `FastaLoader`

The FastaLoader loads reference sequence data in FASTA format and produces tuples that correspond to segments of the reference. These tuples could then be, for example, co-grouped with tuples that result from an imported BAM file of reads aligned to a matching reference sequence in order to compare read with reference bases. For more information on the FASTA format see `http://en.wikipedia.org/wiki/FASTA_format`.

Usage: `reference_bases = load 'hg19.fa' using FastaLoader();`

Output schema:

Field	Type	Description
index_sequence	`chararray`	name of reference fragment (e.g., chromX)
start	`integer`	1-based start position of current reference fragment
sequence	`chararray`	reference bases

5.3 `seqpig.filter`

This module contains various functions that implement Apache Pig's filter UDF interface.

5.3.1 `CoordinateFilter`

The CoordinateFilter filters aligned reads by Samtools regions. See also Section 3.1.4. Note that this filter requires a SAM file header to operate, which can be extracted for example by running `prepareBamInput.sh` script when uploading a BAM file to HDFS.

Usage:

```
DEFINE myFilter CoordinateFilter('input.bam.asciiheader','20:1-44350673');
reads = load 'input.bam' using BamLoader('yes');
reads = FILTER reads BY myFilter(refindex,start,end);
```

Input schema:

Field	Type	Description
refindex	`integer`	reference sequence index
start	`integer`	start position
end	`integer`	end position

5.3.2 `SAMFlagsFilter`

SAMFlagsFilter is a filter that uses SAM flags for filtering. For more example of filtering by SAM flags see Section 3.1.2. Also note the pre-defined set of filters that can be activated by running `run scripts/filter_defs.pig` from within the SeqPig main directory.

Usage:

```
DEFINE ReadUnmapped SAMFlagsFilter('HasSegmentUnmapped');
reads = load 'input.bam' using BamLoader('yes');
mapped_reads = FILTER reads BY not ReadUnmapped(flags);
```

Input schema:

Field	Type	Description
flags	`integer`	SAM flags of read

5.3.3 `BaseFilter`

The BaseFilter is not actually a filter UDF in the Pig sense. Instead, it scans the given set of 2-tuples, consisting of read bases and their base qualities, and replaces all bases that have quality values below a given threshold by "N".

Usage:

```
DEFINE baseFilter BaseFilter('50');
reads = LOAD 'input.fq' USING FastqLoader();
reads_filtered = FOREACH reads GENERATE instrument, run_number, flow_cell_id,
    lane, tile, xpos, ypos, read, qc_passed, control_number, index_sequence,
    FLATTEN(baseFilter(sequence, quality));
```

Input schema:

Field	Type	Description
sequence	`chararray`	read bases
quality	`integer`	base qualities

5.4 `seqpig.stats`

This package contains some UDF's that aim to provide FastQC like statistics for sequence data. They rely on an optimized counter class that maintains counters for discrete range variables (nucleotides, base qualities, etc.) that can be expressed as pairs. For example, one may be interested in counting the number of times base quality 30 appears at position 10 within a given set of reads. This type of counter implements interfaces of Apache Pig that, among other things, allow for in-memory map side aggregation of subresults, which leads to significant runtime improvements due

to a reduction in memory allocation overhead. This optimization comes at the expense of fixing the set of possible values that can be observed (bases, base qualities) and the number of position in question (the readlength). In the rest of the section we therefore assume a given upper bound on the readlength, which we denote by READLENGTH. For implementation details of the counter see `ItemCounter2D.java`.

5.4.1 BaseCounts

The BaseCounts UDF relies on the aforementioned counter class to provide a histogram of the occurrences of each of the five base identifiers ('A', 'C', 'G', 'T' and 'N') over the given set of reads. Note that the output consists of an array of Java long values that are encoded inside a byte array. For convenience one should call the UDF FormatQualCounts to unpack the byte arrays and obtain the original counter values.

Usage:

```
reads = LOAD 'input.fq' USING FastqLoader();
read_seqs = FOREACH reads GENERATE sequence;
base_counts = FOREACH (GROUP read_seqs ALL) GENERATE BaseCounts($1);
formatted_base_counts = FOREACH base_counts GENERATE FormatBaseCounts($0);
```

	Field	Type	Description
Input schema:	*arbitrary*	`chararray`	bases
Output schema:	*long_array_NxM*	`bytearray`	counter values encoded as bytes (N=5 and M is equal to READLENGTH)

5.4.2 FormatBaseCounts

FormatBaseCounts takes the output of BaseCounts and extracts its counter values. For a complete example see above or script `scripts/fast_fastqc.pig`.

Usage:

```
base_counts = FOREACH (GROUP read_seqs ALL) GENERATE BaseCounts($1);
formatted_base_counts = FOREACH base_counts GENERATE FormatBaseCounts($0);
```

	Field	Type	Description
Input schema:	*long_array_NxM*	`bytearray`	counter values encoded as bytes (N=5 and M is equal to READLENGTH)
Output schema:	*pos*	`integer`	position (ranging from 0 to READLENGTH-1)
(bag)	*count1...countN*	`double`	fraction of reads having this base at the given position

5.4.3 FormatGCCounts

FormatGCCounts operates similarly to FormatBaseCounts in the sense that it takes the output of BaseCounts and extracts its counter values. Instead of considering all possible bases (including 'N'), it instead only considers the GC content at each position of the reads.

Usage:

```
reads = LOAD 'input.fq' USING FastqLoader();
read_seqs = FOREACH reads GENERATE sequence;
base_counts = FOREACH (GROUP read_seqs ALL) GENERATE BaseCounts($1);
formatted_gc_counts = FOREACH base_counts GENERATE FormatGCCounts($0);
```

	Field	Type	Description
Input schema:	*long_array_NxM*	`bytearray`	counter values encoded as bytes (N=5 and M is equal to READLENGTH)
Output schema:	*pos*	`integer`	position (ranging from 0 to READLENGTH-1)
(bag)	*GC*	`double`	fraction of reads having 'G' or 'C' at this position

5.4.4 AvgBaseQualCounts

The AvgBaseQualCounts UDF relies on the aforementioned counter class to provide a histogram of the average base quality (rounded to next integer) over the given set of reads (average taken over the positions within the read). Note that the output consists of an array of Java long values that are encoded inside a byte array. For convenience one should call the UDF FormatAvgBaseQualCounts to unpack the byte arrays and obtain the original counter values.

Usage:

```
reads = LOAD 'input.fq' USING FastqLoader();
read_seq_qual = FOREACH reads GENERATE quality;
avgbase_qual_counts = FOREACH (GROUP read_seq_qual ALL) GENERATE
    AvgBaseQualCounts($1.$0);
formatted_avgbase_qual_counts = FOREACH avgbase_qual_counts GENERATE
    FormatAvgBaseQualCounts($0);
```

	Field	Type	Description
Input schema:	*arbitrary*	`chararray`	base qualities
Output schema:	*long_array_NxM*	`bytearray`	counter values encoded as bytes (N is the number of different quality values and M is 1 in this case)

5.4.5 FormatAvgBaseQualCounts

FormatAvgBaseQualCounts takes the output of AvgBaseQualCounts and extracts its counter values. For a complete example see above or script `scripts/fast_fastqc.pig`.

Usage:

```
avgbase_qual_counts = FOREACH (GROUP read_seq_qual ALL) GENERATE
    AvgBaseQualCounts($1.$0);
formatted_avgbase_qual_counts = FOREACH avgbase_qual_counts GENERATE
    FormatAvgBaseQualCounts($0);
```

	Field	Type	Description
Input schema:	*long_array_NxM*	`bytearray`	counter values encoded as bytes (N is the number of different quality values and M is 1 in this case)
	pos	`integer`	position (always 0 in this case)
Output schema:	*mean*	`double`	avg. base quality, averaged over the set of reads
(bag)	*stdev*	`double`	standard deviation of average base quality over the set of reads
	qual1...qualN	`double`	number of occurrences of each (average) quality

5.4.6 BaseQualCounts

Usage:

```
reads = LOAD 'input.fq' USING FastqLoader();
qualities = FOREACH reads GENERATE quality;
base_qual_counts = FOREACH (GROUP qualities ALL) GENERATE BaseQualCounts($1);
formatted_base_qual_counts = FOREACH base_qual_counts GENERATE
    FormatBaseQualCounts($0);
```

	Field	Type	Description
Input schema:	*arbitrary*	`chararray`	base qualities
Output schema:	*long_array_NxM*	`bytearray`	counter values encoded as bytes (N is the number of different quality values and M is the READLENGTH)

5.4.7 FormatBaseQualCounts

FormatBaseQualCounts takes the output of BaseQualCounts and extracts its counter values. For a complete example see above or script `scripts/fast_fastqc.pig`.

Usage:	`base_qual_counts = FOREACH (GROUP qualities ALL) GENERATE BaseQualCounts($1);`<br>`formatted_base_qual_counts = FOREACH base_qual_counts GENERATE FormatBaseQualCounts($0);`		
	Field	Type	Description
Input schema:	*long_array_NxM*	`bytearray`	counter values encoded as bytes (N is the number of different quality values and M is the READLENGTH)
Output schema: (bag)	*pos*	`integer`	position (ranging from 0 to READLENGTH-1)
	mean	`double`	avg. base quality at this position, averaged over the set of reads
	stdev	`double`	standard deviation of base quality at this position
	qual1...qualN	`double`	number of occurrences of each read quality at this position

5.5 seqpig.pileup

The pileup operation implemented in SeqPig basically consists of two stages. First, each read is inspected and pileup relevant output is produced for each of the positions of the reference that it "spans". Note that this may be also more or less than the readlength, depending if there were insertions or deletions. Then the output of this first stage is grouped by (chromosome, position) and pileup output from different reads affecting the same reference position is merged. The output should be the same that can be obtained by an operation of Samtools mpileup.

The last UDF in this section (BinReadPileup) increases parallelism by adding a pre-processing step that first partition reads into bins by their alignment position and then calls the two other UDF's in each of the bins. Fore more details see Section 3.1.8 and for a complete example see script `scripts/pileup2.pig`.

5.5.1 ReadPileup

The ReadPileup UDF performs the steps of the first stage described above and operates on single reads that are passed with a subset of their fields. It produces a bag of tuples as output.

Usage:	`reads = load 'input.bam' using BamLoader('yes');`<br>`read_pileup = FOREACH reads GENERATE ReadPileup(read, flags, refname, start,`<br>`   cigar, basequal, attributes#'MD', mapqual), start, flags, name;`<br>`read_pileup = FILTER read_pileup BY $0 is not null;`<br>`pileup = FOREACH read_pileup GENERATE flatten($0), start, flags, name;`<br>`grouped = GROUP pileup BY (chr, pos);`<br>`result = FOREACH grouped {`<br>`   tmp = FOREACH pileup GENERATE refbase, pileup, qual, start, (flags/16)%2, name;`<br>`   tmp = ORDER tmp BY start, $4, name;`<br>`   GENERATE group.chr, group.pos, PileupOutputFormatting(tmp, group.pos);`<br>`}`

	Field	Type	Description
Input schema:	*sequence*	`chararray`	read bases
	flag	`integer`	SAM flags
	chr	`chararray`	chromosome / reference name
	position	`integer`	position of read
	cigar	`chararray`	CIGAR string
	basequal	`chararray`	base qualities
	md	`chararray`	MD tag
	mapqual	`integer`	mapping quality
Output schema: (bag)	*chr*	`chararray`	chromosome / reference name
	position	`integer`	position (on reference)
	refbase	`chararray`	base on reference (if known, else null)
	pileup	`chararray`	pileup string for this read
	qual	`chararray`	base quality/ies for this read (if applicable, else null)

5.5.2 `PileupOutputFormatting`

The PileupOutputFormatting UDF performs the second stage of the pileup, which is the merging of the output coming from different reads for the same reference position. Note that the UDF assumes that input is passed in the order of reference position. For usage see the example code above.

	Field	Type	Description
Input schema:	*pileup*	`bag`	bag of tuples produced by ReadPileup, grouped by position
	position	`integer`	reference position this pileup data originates from
Output schema: (bag)	*refbase*	`chararray`	reference base
	count	`integer`	number of reads (depth)
	pileup	`chararray`	pileup string
	qual	`chararray`	base qualities

5.5.3 `BinReadPileup`

This UDF aims to increase parallelism during the pileup computation by partitioning reads into bins by their alignment position and then calling the two previous UDF's in each of the bins. Note that each bin corresponds to a half-interval over the reference position of some reference segment (chromosome). The bin size (500 bases in the example code below) is essentially arbitrary but should be larger than the read length (plus maximum insertion size). For a complete example see script `scripts/pileup2.pig`.

Usage:	`reads = load 'input.bam' using BamLoader('yes');`<br>`crossing_Reads = FILTER reads BY start/500!=end/500;`<br>`crossing_reads = FOREACH crossing_Reads GENERATE read, flags, refname, start, cigar, basequal, attributes#'MD', mapqual, name, end/500;`<br>`all_reads = FOREACH reads GENERATE read, flags, refname, start, cigar, basequal, attributes#'MD', mapqual, name, start/500;`<br>`input_reads = UNION crossing_reads, all_reads;`<br>`grouped_reads = GROUP input_reads BY (refname, $9);`<br>`pileup = FOREACH grouped_reads GENERATE BinReadPileup(input_reads,group.$1*500,(group.$1+1)*500);`<br>`pileup = FILTER pileup BY $0 is not null;`<br>`result = FOREACH pileup GENERATE flatten($0);`<br>`result = ORDER result BY chr, pos;`

	Field	Type	Description
Input schema:	*reads*	`bag`	bag of read tuples (each adhering to the input schema of ReadPileup)
	left_pos	`integer`	left border position of bin (inclusive)
	right_pos	`integer`	right border position of bin (exclusive)
Output schema: (bag)	*chr*	`chararray`	chromosome / reference name
	pos	`integer`	position (on reference)
	refbase	`chararray`	base on reference
	count	`integer`	number of reads (coverage)
	pileup	`chararray`	pileup string for this position
	qual	`chararray`	base quality/ies for this read
